# Differences in realized access to healthcare among newly arrived refugees in Germany: results from a natural quasi-experiment

**DOI:** 10.1186/s12889-020-08981-2

**Published:** 2020-06-03

**Authors:** Judith Wenner, Kayvan Bozorgmehr, Stella Duwendag, Kristin Rolke, Oliver Razum

**Affiliations:** 1 Department of Epidemiology & International Public Health, School of Public Health, Bielefeld University, P.O. Box 10 01 31, 33501 Bielefeld, Germany; 2 Department of Population Medicine and Health Services Research, School of Public Health, Bielefeld University, P.O. Box 10 01 31, 33501 Bielefeld, Germany

**Keywords:** Refugees, Access to healthcare, Natural quasi-experimental design, Claims data

## Abstract

**Background:**

Germany has a statutory health insurance (SHI) that covers nearly the entire population and most of the health services provided. Newly arrived refugees whose asylum claim is still being processed are initially excluded from the SHI. Instead, their entitlements are restricted and parallel access models have been implemented. We assessed differences in realized access of healthcare services between these access models.

**Methods:**

In Germany’s largest federal state, North Rhine-Westphalia, two different access models have been implemented in the 396 municipalities: the healthcare voucher (HcV) model and the electronic health card (eHC) model. As refugees are quasi-randomly assigned to municipalities, we were able to realize a natural quasi-experiment including all newly assigned refugees from six municipalities (three for each model) in 2016 and 2017. Using claims data, we compared the standardized incidence rates (SIR) of specialist services use, emergency services use, and hospitalization due to ambulatory care sensitive conditions (ACSC) between both models. We indirectly standardized utilization patterns first for age and then for the sex.

**Results:**

SIRs of emergency use were higher in municipalities with HcV (ranging from 1.41 to 2.63) compared to emergency rates in municipalities with eHC (ranging from 1.40 to 1.71) and differed significantly from the expected rates derived from official health reporting. SIRs of emergency and specialist use in municipalities with eHC converged with the expected rates over time. There were no significant differences in standardized hospitalization rates for ACSC.

**Conclusion:**

The results suggest that the eHC model is slightly better able to provide refugees with SHI-like access to specialist services and goes along with lower utilization of emergency services compared to the HcV model. No difference between the models was found for hospitalizations due to ACSC. Results might be slightly biased due to incompletely documented service use and due to (self-) selection on the level of municipalities with municipalities interested in facilitating access showing more interest in joining the project.

## Background

According to the aim of universal health coverage (UHC) promoted by the World Health Organization, a health system should “provide all people with access to needed health services” (p.6) [1]. Thus, healthcare access should be based on need rather than other factors (e.g. residence status, access model). To achieve equal access for equal need, many countries, including Germany, have established a statutory health insurance (SHI). Germany’s SHI is organized according to the principle of solidarity with contributions adjusted to financial means, shared risk-pooling and tax-financed transfers to cover fees of the unemployed. Membership in the SHI is mandatory with only a few exceptions (for affluent persons, public officials and self-employed), and these groups have to prove any other health insurance coverage instead [2, 3].

Newly arrived refugees whose asylum claim is still being processed – subsequently called “refugees” – are explicitly excluded from the SHI in Germany during the first months of their stay (at the time of our study usually 15 months). Instead, they fall under the federal asylum seekers' benefits act (ASBA) according to which their healthcare entitlements are restricted. According to the entitlement restrictions, refugees are entitled to treatment for acute illness and pain, pregnancy and birth, as well as for officially recommended vaccination and medically necessary check-ups only (§4 ASBA). Necessary treatment in all other cases, e.g. psychotherapy, is only granted on a case-by-case basis (§6 ASBA). In addition, and in the focus of this article, the ASBA establishes parallel access models. These parallel access models for refugees fall into the responsibility of the federal states and municipalities to which newcomers are assigned to. As of today, two (parallel) access models are implemented on the local level: the healthcare voucher (HcV) model or the electronic health card (eHC) model. The HcV model was the only model for many years. Before accessing services, refugees need to obtain paper-based HcVs from the local social welfare office (SWO). The HcVs are valid for one quarter of the year and are handed over to the healthcare providers of their choice – usually to the general practitioner (GP) – upon visit. Subsequently, providers issue bills to the SWOs that refund the providers. Referrals from GPs to many specialists or for hospitalization undergo a legal review by non-medically trained staff of the SWO. The only exception is emergency treatment for which no HcV is needed.

The eHC model is a rather recent model (first implemented in 2005) and was developed with the aim of facilitating access to healthcare services for refugees [4, 5]. Some federal states and municipalities stopped using the HcV model and instead issued eHCs that are utilizable just like the standard eHC issued by the German SHI. They are valid for at least 15 months and are kept by the patients for repeated use. Refunding of services is organized by the SHI and the case-by-case reviews for referrals were largely abolished. The major difference to SHI-cards – which makes it a parallel access model – is that refugees do not become members of the SHI. Thereby, they are excluded from risk-pooling, and the legal entitlement restrictions previously mentioned stay in place [6–8].

There is empirical and theoretical [9–12] work stressing the importance of health system organization for realized access to healthcare – which we equate here to actual use or utilization of healthcare services following Ronald M. Andersen’s definition [9]. However, little is known on the importance of these local access models on refugees’ realized access to healthcare in Germany. It has been shown in qualitative studies that the HcVs may be perceived as a barrier to outpatient care by refugees [13]. Similarly, in a survey among internal medicine specialists in Germany, a considerable proportion reported not being familiar with the HcV regulations and the ASBA [14]. Quantitative studies reported high unmet needs among adult refugees [15], high odds of emergency department use and avoidable hospitalizations among refugee children compared to non-refugees [16, 17]. So far, only one study directly compared healthcare use of refugees using different access models by means of a regional survey, showing an underuse of outpatient healthcare among refugees using the HcV model when compared to refugees who were using the eHC model [18]. However, from other studies comparing health service use among regularly insured patients in Germany to refugee patients, even refugees that have been issued eHCs less frequently used outpatient care, especially specialized care and mental health services [19, 20]. At the same time, higher rates of avoidable hospitalizations compared to regularly SHI-insured were reported [20]. Whether the differences in utilization between refugees and regularly insured persons are related to the access model, the entitlement restrictions, or other access barriers (e.g. language), has not been analysed in these studies.

The aim of our research was to isolate the effects of the access model from other determinants of realized access among refugees. The research question guiding our study was thus whether realized access to healthcare differs between the two local access models (eHC and HcV).

Methods and results will be reported in line with the STROBE – statement of reporting of results from observational studies [21].

## Methods

We chose the federal state of North Rhine-Westphalia (NRW), the largest federal state, for our empirical study as it is the only one with a mix of access models implemented within the same state legislation in a sufficiently large number of municipalities [22]. The detailed study protocol has been published elsewhere [23]. We considered our study to be a natural quasi-experiment because refugees are obliged to reside in particular federal state and are subsequently assigned to municipalities shortly after their arrival. Assignment within NRW takes place according to population and area size of the municipalities [24]. As the 396 municipalities in NRW used two different access models, the dispersal also assigns refugees to a given model. As researchers we were thus not involved in the assignment to the healthcare models as exposure of interest – a major characteristic of natural experiments [25]. However, assignment in experiments has to be completely at random [26]. The assignment in our study was only quasi-random as refugee families (and not individuals) are assigned together, and severe illnesses or special accommodation needs might influence the assignment in some cases [24]. Thus, we deemed it to be a mixture of two types of study designs, quasi-experiment and natural experiment [27], and considered the term natural quasi-experiment to be adequate here. Given the small number of municipalities and the short timeframe, our study is explorative.

We included six municipalities – three using each of the models – in our study to reach the necessary sample size [23]. In all municipalities, the local SWO had to agree to participate in our project. In the six municipalities, we included all refugees that were entitled to using the respective models during the study period of seven quarters (2nd quarter of 2016 (=Q1) until 4th quarter of 2017 (=Q7)). They joined the sample when assigned to one of the six municipalities and left the sample after 15 months of stay in Germany, or when their asylum claim had been accepted (open cohort). The sample size therefore mainly reflected the number of refugees arriving in Germany and being assigned to a municipality shortly after arrival. Refugees accused of not cooperating with the migration authorities (Art. 1a ASBA) are prevented from joining the SHI and stayed in the sample even longer.

Claims data was collected retrospectively from the responsible local data owner which was either the SWO or the SHI. Data contained information on all services reimbursed by the SWO or the SHI. It included date of use (quarter of the year), diagnoses (according to the German version of the 10th revision of the international classification of diseases (ICD-10-GM)), type of provider (GP, specialist, department in hospital) and type of service (emergency or regular). In addition, the SWOs or SHIs provided us with aggregated information on the age and sex distribution of persons registered in the municipalities for each quarter.

To be able to compare realized access – operationalized as utilization of healthcare services – between the two models, we selected three indicators (two process and one outcome indicator). We hypothesized lower rates of specialist outpatient service use (process indicator I) in HcV municipalities due to the additional efforts for patients associated with referrals when using the HcV (exposition) compared to the eHC (control). In addition, we assumed delays in or deferral of treatment which results in higher emergency service use (process indicator II) and higher rates of ambulatory sensitive hospitalizations (outcome indicator I).

To calculate standardized incidence rates (SIRs) of **specialist use** (process indicator I), we included only the first visit in the quarter and excluded dentists (as documentation was inconsistent), gynaecologists (as standardization of combined age-sex information was not feasible) and psychotherapists (as utilization was highly confounded by the local availability of service providers) from further analysis.

For the analysis of **emergency service use** (process indicator II), we included all visits to the emergency department or to the emergency services of the outpatient physicians. When calculating SIRs, we considered only the first use of emergency service per quarter.

For the analysis of inpatient service use, we included all hospitalizations for which the major diagnosis was due to an **ambulatory care sensitive condition** (ACSC) (outcome indicator I). ACSC are defined as “conditions for which good outpatient care can potentially prevent the need for hospitalization, or for which early intervention can prevent complications or more severe disease” (p.1) [28]. International catalogues of ACSC have been adapted to the German context. The final version includes 258 ICD codes [29]. For hospitalizations in children’s wards, we used a slightly modified list of codes [30]. We had to exclude conditions related to gynaecological or psychiatric diseases as ICD codes were not available for many of these hospitalizations. In addition, we had to exclude conditions for which the categorization as ACSC depended on the fifth digit as our data consisted of four-digit ICD codes only.

We calculated crude utilization rates for the whole study period using the total number of person-quarters (PQs; the person-time unit that could be calculated with the data available) as denominator. Subsequently, we calculated age- and sex-adjusted SIR for each quarter (1), controlling for confounding that persisted after quasi-random assignment. The sex variable was dichotomous. The age-variable was categorical with four age groups (0–14, 15–25, 26–65, > 65) in outpatient data and nine age groups (0–14, 15–17, 18–25, 26–35, 36–45, 46–55, 56–65, 66–79, ≥80) for inpatient data. We assessed difference in age and sex distribution between observational groups and tested its significance using chi^2^-test (with *p* < 0.05 considered significant).

1 $$ {SIR}_q=\frac{\sum ob\mathrm{s} erved\ cases\ }{\sum expected\ cases} $$

2 $$ E=\sum \limits_{i=1}^k\left({n}_i\ast {R}_i\right) $$

SIRs were calculated using indirect standardization whereby incidence rates are adjusted using age- (or sex-) specific incidence rates of a standard population and applying them to the age (or sex) distribution of the study population. The number of expected cases (E) in the study population results from the combination of the size of each stratum in the study population (n) with the incidence rates from the standard population (R, here called expected rates) (2). We had to use different data sources to obtain the necessary expected rates for the three outcomes, but they were all taken from official health reporting on utilization of healthcare services among the resident population in Germany. For expected rates of specialists’ service use, we drew on two large national health surveys (the German Health Interview and Examination Survey for Adults [31] and the German Health Survey for Children and Adolescents [32]) conducted by the Robert Koch-Institute. Expected rates for emergency service use were taken from the health report of a large SHI [33]. Sex-specific rates were not included in the data set. For emergency use, we thus conducted age standardization and excluded emergency use due to gynaecological conditions. For standardization of ACSC, we used the statistics of hospital diagnosis [34] and the population statistics [35] of the federal office of statistics.

We calculated 95%-Confidence (CI) intervals for SIRs, assuming that our estimates (that is, the expected number of cases) approximately followed a Poisson distribution. Given the problems of comparing indirectly adjusted rates between groups [36, 37] our analyses had an explorative character. All analyses were conducted using Stata version 15 [38]. For standardization and CI calculation we used the *istdize* command [39]. Tables and figures in the results were created using Microsoft Excel 2013.

Missing information on one of the outcomes or confounders led to the exclusion for the analysis of this outcome or standardization, but not for other outcomes. One municipality had to be excluded from the analysis of hospitalization as the reporting of ICD codes was incomplete.

For sensitivity analysis, we also calculated adjusted SIRs by multiplying the SIR with the crude rate of the respective standard populations. With these adjusted rates, we calculated simple moving averages for a period of three quarters, thereby accounting for dependency of quarterly rates. In addition, we repeated all analyses for the six municipalities separately to explore effects of clustering (by municipalities) within the groups. Furthermore, we compared utilization rates (including all cases) and incidence rates (counting only if any case was documented per person and quarter). Results from sensitivity analysis will be reported only briefly.

## Results

**Fig. 1:**
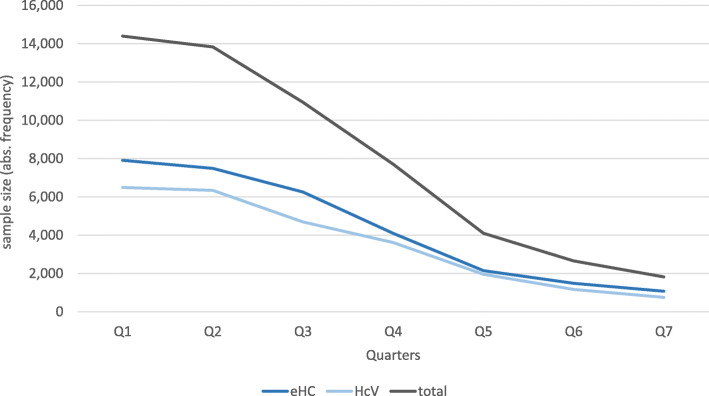
Development of the study sample over time (2016–2017)

During the study period of seven quarters in 2016/17, our study sample comprised 14,400 persons at maximum in April 2016 and 1822 persons at minimum in December 2017. During the whole study period, this amounted to 55,452 PQ in the whole sample; 30,451 PQs accrued in eHC municipalities and 25,001 PQs in HcV municipalities. Our average study population corresponded to 5.7% of all refugees using any of the two models in NRW at the end of 2016 [40](c.f. Figure 1).

On average, 36.2% of the sample were women. The share of women in the comparison groups differed slightly, but significantly in 5 out of 7 quarters. The majority of persons in the sample were adults aged between 25 and 64 years, followed by an approximately equal share of around 20% children (0–14 years) and youth (15–24 years). Only around 1% of the sample were 65 years or older. The age distribution differed significantly between both groups in 3 out of the 7 quarters (c.f. Table 1).

**Table 1:** Study population according to access model, age and sex

Access model		Quarters of the year (2/2016 to 4/2017)						
**eHC**		**Q1**	**Q2**	**Q3**	**Q4**	**Q5**	**Q6**	**Q7**
	**age (in % of n)**							
	**0–14**	27.2	28.0	27.5	25.7	24.7	19.7	20.1
	**15–24**	26.7	26.7	26.9	26.5	24.5	26.8	26.5
	**25–64**	45.4	44.5	44.8	46.8	49.6	52.4	52.3
	**> 65**	0.7	0.8	0.8	1.0	1.2	1.1	1.0
	**sex (in % of n)**							
	**female**	34.8	35.6	35.9	35.8	36.3	31.7	31.1
	**total (n)**	**7909**	**7491**	**6252**	**4088**	**2149**	**1488**	**1074**
**HcV**		**Q1**	**Q2**	**Q3**	**Q4**	**Q5**	**Q6**	**Q7**
	**age (in % of n)**							
	**0–14**	27.9	28.7	27.4	26.9	26.5	25.4	24.9
	**15–24**	27.0	26.6	27.6	27.7	27.4	26.8	25.4
	**25–64**	44.3	43.9	44.2	44.2	44.4	46.2	48.0
	**> 65**	0.8	0.8	0.8	1.2	1.6	1.5	1.7
	**sex (in % of n)**							
	**female**	37.2	37.8	38.2	36.9	37.4	39.8	39.2
	**total (n)**	**6491**	**6342**	**4687**	**3614**	**1951**	**1168**	**748**
**Total (n)**		**14,400**	**13,833**	**10,939**	**7702**	**4100**	**2656**	**1822**
**X**^**2**^**-test (age)**		0.590	0.827	0.888	0.118	0.007*	0.001*	0.044*
**X**^**2**^**-test (sex)**		0.002*	0.007*	0.017*	0.294	0.450	0.000*	0.000*

*p < 0.05

### Specialist service use

There was a total number of 30,073 cases of specialist use in the whole study period (excluding gynaecologist, dentists and psychotherapists). The crude utilization rate in municipalities with HcV was lower (446/1000 PQ) compared to the rate in municipalities with eHC (622/1000 PQ). The share of specialist visits in municipalities with eHC was significantly larger than in municipalities with HcV (40.5% vs. 31.5%; *p* < 0.001) where the majority of outpatient visits were GP visits (51.5%, compared to 41.6% in municipalities with eHC; *p* < 0.001). The specialist visited was not specified for *n* = 353 (eHC: 0.8%) and *n* = 810 (HcV: 2.3%) cases (c.f. Table 2).

**Table 2:** Overview of outpatient service use

	eHC			HcV		
	n	%	crude (utilization) rate (per 1000 PQ)	n	%	crude (utilization) rate (per 1000 PQ)
**Specialists (excluding all mentioned below)**	18,931	40.5	622	11,142	31.5	446
**GP (adults and children)**	19,434	41.6	638	18,123	51.3	725
**Gynaecologists**	4287	9.2	141	3016	8.5	121
**psychiatrist or psychotherapists**	429	0.9	14	223	0.6	9
**Dentists**	3269	7.0	107	2012	5.7	80
**not specified**	353	0.8	12	810	2.3	32
**Total (n)**	46,703	100		35,326	100	
Of these emergency cases	3409	7.3	112	2761	7.8	110
**Total (PQ)**	30,451			25,001		

*eHC* Electronic health card, *HcV* Health care voucher, *n* absolute number of visits; *PQ* Person-quarters; % = share of total (n).

**Fig. 2:**
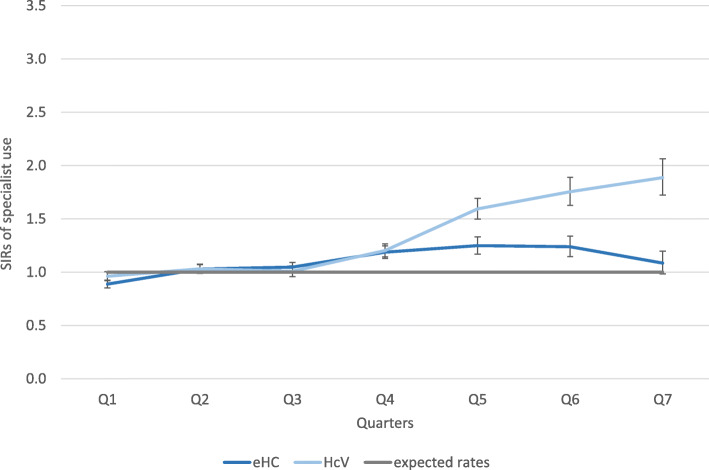
Quarterly age-adjusted SIRs of specialist service use (and 95%-CIs; CIs not overlapping with the standard rates (=1) indicate significant differences (at 5% level))

The SIRs for age and sex in municipalities with eHC did not differ markedly from the expected rates. They were approximately 1 in all quarters and only slightly but significantly larger (with the 95%-CI not including 1) in three quarters. In municipalities with HcV, SIRs did not differ from the expected rates in three quarters, but diverted significantly in the remaining four quarters indicating higher utilization compared to the standard population (c.f. Figure 2, SIRs for sex standardization approximately equal to age-adjusted SIRs and therefore not shown). There is a clear time trend with SIRs increasing in municipalities with HcV over time while they increase only slightly in municipalities with eHC.

Sensitivity analysis revealed a higher share of repeated visits to (the same and different) specialists by the same person in municipalities with eHC compared to municipalities with HcV. Calculation of moving averages did not change the results and the observed time trend considerably. SIRs for the single municipalities were in line with the pooled results – except for one municipality with HcV where SIRs were considerably lower than expected rates.

### Emergency service use

**Fig. 3:**
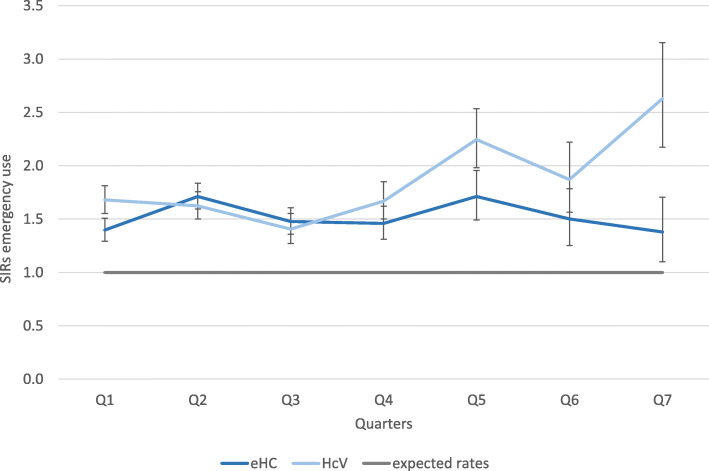
Quarterly age-adjusted SIRs of emergency service use (and 95%-CIs; CIs not overlapping with the standard rates (=1) indicate significant differences (at 5% level))

During the study period of 21 months, 6170 emergency cases were recorded in the six municipalities. This constituted a share of 7.3% of all outpatient encounters in municipalities with eHC and a share of 7.8% in municipalities with HcV. The crude utilizations rates were nearly equal in both groups (112 and 110 per 1000 PQ). There was no missing information on whether an outpatient encounter was an emergency case or not (c.f. Table 2). Calculation of SIRs showed higher emergency utilization in municipalities with HcV compared to municipalities with eHC. SIRs were significantly higher compared to expected rates for both groups (95%-CIs not including 1), but SIRs in municipalities with eHC decreased over time and thereby converged with the expected rates over time (c.f. Figure 3.). In municipalities with HcV, SIRs increased over time and diverged from the expected rates during the study period.

There were no sex-specific rates available for the standard population. However, excluding emergency use for gynaecological conditions helped to control for the major sex difference observed in the standard population [33]. This did not largely change the differences observed for age-standardized rates. Quarterly moving averages of the adjusted SIRs showed similar patterns. SIRs were still higher compared to the standard population and on average higher in municipalities with HcV. When considering moving averages of SIRs, the time trend proved to be very obvious with nearly constant SIRs in municipalities with eHC and a marked increase in municipalities with HcV. Only few emergency cases were repeated visits by the same person (8.3%) and therefore including or excluding them led to only minor changes in crude utilization rates. Calculation of SIRs for single municipalities led to similar results for the sample of municipalities with eHC. Results for municipalities with HcV are largely influenced by high SIRs in one municipality.

### Hospitalization for ACSC

**Fig. 4:**
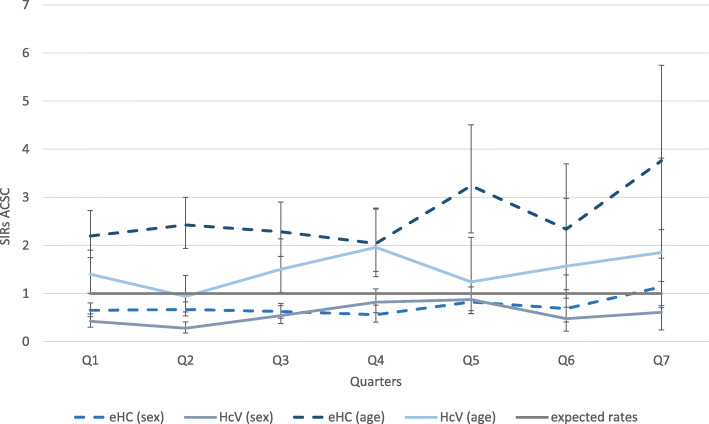
Quarterly age- and sex-adjusted SIRs of hospitalization for ACSC (and 95%-CIs; CIs not overlapping with the standard rates (=1) indicate significant differences (at 5% level))

We registered a total number of 465 ACSC cases in our sample – excluding the municipality with poor reporting of hospital diagnoses. This corresponds to crude rates of 10/1000 PQs in municipalities with eHC model and to 7/1000 PQs in municipalities with HcV model. The sex-adjusted SIRs did not differ manifestly between both groups while the age-adjusted SIR differed slightly with higher SIRs in municipalities with eHC. However, in all quarters and both groups age-adjusted SIRs were higher, and the sex-adjusted SIRs were lower than the expected rates, suggesting that combined age-sex-standardization would have been necessary, but was not possible with our data (c.f. Figure 4).

Excluding repeated ACSC cases for sensitivity analysis did not significantly alter the quarterly SIRs. Calculating moving average of the adjusted SIRs led to further convergence of the rates. SIRs for the single municipalities were similar to the pooled SIRs for each group.

## Discussion

We have explored the difference in realized access to healthcare among refugees in six municipalities in Germany’s largest federal state of NRW, comparing two currently implemented local access models – eHC and HcV. Our results showed that there are differences between the groups with regard to realized access to specialist and emergency services. In municipalities with eHC, SIRs for these two outcomes converged with the expected rates (derived from official health reporting), while it diverged in municipalities with HcV. These differences were less obvious or even absent in the initial comparison of the crude rates calculated without adjustment for age or sex distribution and for the whole study period. This is at least partly explained by slightly different age and sex distributions of the samples. It also shows that the different utilization patterns in municipalities with eHC and HcV over time are evened out by overall utilization rates.

Based on the observed differences in SIRs we assume that the eHC model is more likely to provide refugees with SHI-like access to outpatient care compared to the HcV model. In addition, the lower SIR of emergency cases in municipalities with eHC has clear benefits for the patients, the providers and the municipalities financing the services.

The divergence of the SIRs in municipalities with HcV for specialist and emergency care are parallel to the decrease of the sample size and the decrease in newly arrived refugees in Germany respectively. Part of the effect might be explained statistically by a decreasing study population. However, the study population decreased similarly in municipalities with eHC where the SIRs remain rather constant or even decreased slightly over time. The increasing SIRs in municipalities with HcV might therefore hint at model-specific differences in utilization. One possible explanation is that the initially high number of incoming refugees led to a delay in utilization among refugees in municipalities with HcV and subsequently to higher utilization in the latter quarters once the number of newly arriving refugees had decreased. If this delay was directly related to the issuing and utilization of HcVs (which cannot be proven with our data), the eHC model might be better able to provide access even in times of high numbers of incoming refugees. This should be further explored.

We did not find consistent differences between the two models for hospital admissions due to ACSC as suggested by empirical studies on hospitalizations of refugees in Germany [20, 30]. This is at least partly due to the fact that the data did not allow for combined standardization making the interpretation of the SIRs difficult in our study. Given the age distribution of our sample (predominantly young adults) the rates were rather high. On the contrary, given the sex distribution (nearly two thirds male and ACSC related to delivery and reproductive health excluded) the ACSC rates were slightly lower compared to the standard rates. As this effect is present in both samples in a similar way (eHC and HcV), we see no significant difference between the models. However, the small absolute number of cases in both groups made finding significant differences unlikely.

Given the absence of differences for ACSC and the advantages of the eHC for outpatient use, the results should help to overcome wrong concerns of opponents of the eHC model who had anticipated that it might lead to an inadequate overuse of healthcare services among refugees [19, 41]. Both models are implemented in other federal states of Germany in a similar way. Thus, the advantages of the eHC model for utilization of outpatient and emergency care we identified are likely to apply in other federal states where the eHC model is introduced or even provide arguments of why it should be introduced. Beyond the immediate implementation of the access models in Germany, the results hint at the advantage of including newly arrived refugees in the standard care model (instead of creating parallel access models) and using digital patient records (instead of paper-based).

An important strength of our study is that it shows the opportunities of using claims data and the feasibility of a natural quasi-experiment in this case. We were able to reach a large sample size without having to recruit newly arrived refugees for a potentially stressful and complex primary data collection [42]. There was no risk of exclusion due to difficulties in communication or recruitment. The composition of the sample with regard to age and sex reflected reporting by the Federal Office of Migration and Refugees on newly arriving refugees in 2016 and 2017 [43]. In addition, our approach allowed us to isolate the effect of the local access models. Our results showed that under restricted entitlements, the access model is associated with differences in specialist and emergency service use, but not with hospitalizations due to an ACSC. The differences attributable to the access model were smaller than differences found in comparisons of groups with restricted entitlements and regular SHI-like access [19, 20, 44]. We therefore assume that the entitlement restrictions for refugees exert higher influence on realized access than the local access model used.

Besides these results and strengths of our study, there are some limitations. We used the age and sex distributions as an approximation for need and controlled for confounding but need itself was not further measured in our study. We thus know little about the individual need and experiences when accessing care or the pathways to healthcare utilization. We also could not control for confounding by family relations in our sample. Families are assigned together and these relationships are not documented in our data [24]. Additionally, we identified potential confounders on municipal level (e.g. available health system infrastructure) that we were not able to control for in our analysis. We were aware of these limitations and embedded our quantitative study – as suggested in literature on methodological aspects of natural experiments [25] – in a mixed-methods approach which included qualitative interviews with SWO staff, healthcare providers, social workers and refugees themselves. The results from these interviews informed our research continuously and support our quantitative findings. Both – local actors and refugee patients – reported advantages of the eHC when accessing healthcare while stressing the importance of other aspects that were present in both models like the entitlement restrictions, the dependency on support by social workers or the lack of funding for interpreters [45].

As we included all refugees in the selected municipalities, selection bias on the level of individuals can be excluded. However, on the level of municipalities, those interested in facilitating access to healthcare for refugees were more likely to participate in the project – especially in municipalities with HcV. They facilitated access by handing out new HcVs in case of loss, sending them automatically to accommodation centres at the beginning of each quarter or supported refugees in making doctor’s appointments [45]. This might have led to the underestimation of the differences in our outcomes.

We were also aware of more complex or multidimensional concepts of access that do not equate utilization with access [46–48]. However, we were unable to operationalize these concepts with our data. Similarly, we had to analyse quarterly utilization rates (instead of global or annual rates) as the exact age and sex distribution was only available for the quarters of the year. Our results also apply only to utilization within the regular health system refunded by the SWOs. Healthcare use in informal settings or paid out of pocket could not be covered by our study. There are several other constrains when using claims data in general and also specifically to claims data in the German context [49]. We have thus refrained from detailed analysis of diagnoses or treatment. We also discussed questions of plausibility and validity with the data owners to better understand the provided data. Still, we could not preclude minor information bias due to wrong copying of names from HcVs or wrong coding of diagnoses by the healthcare providers or data owners.

Lastly, as we repeatedly compared SIRs for the same outcomes using the same data, we ran a risk of multiple testing. However, as the comparison of rates and their differences was explorative, we did not accept or reject hypotheses based on our data.

## Conclusion

Our results provide evidence for an, albeit small, advantage of the eHC model to provide refugees with SHI-like access to (outpatient) healthcare services and to reduce utilization of emergency services and no difference for inpatient care compared to the HcV model. Remaining differences in rates of realized access between newly arrived refugees (using either the eHC or the HcV model) and regularly insured persons are likely to depend on other factors (e.g. entitlement restrictions) present in both models. Further analysis, ideally using direct standardization or individual level data, should include more municipalities to reduce selection bias and cooperate with them to improve documentation right from the beginning.

## Data Availability

The data that support the findings of this study are available from the municipalities and the statutory health insurance but restrictions apply to the availability of these data, which were used under an agreement between the authors and the municipalities/statutory health insurance for the current study, and so are not publicly available. Data are however available from the authors upon reasonable request and with permission of the municipalities and the statutory health insurance.

## Abbreviations

ACSC: Ambulatory care sensitive conditions
ACSH: Ambulatory care sensitive hospitalizations
ASBA: Asylbewerberleistungsgesetz/Asylum Seekers' Benefits Act
CI: Confidence Intervals
eHC: Electronic health card
GP: General Practitioner
HcV: Health care voucher
NRW: Nordrhein-Westfalen/North Rhine-Westphalia
PQ: Person-Quarters
Q: Quarter
SHI: Statutory Health Insurance
SIR: Standardized Incidence Rates
SWO: Social Welfare Office
UHC: Universal Health Coverage
